# Supplementary material for: Two forms of death in ageing Caenorhabditis elegans
Source: Nat Commun. 2017 May 23;8:15458. doi: 10.1038/ncomms15458 (PMC5457527; doi:10.1038/ncomms15458)
Supplement: Supplementary Information — Supplementary Figures, Supplementary Tables, Supplementary References. [file ncomms15458-s1.pdf]

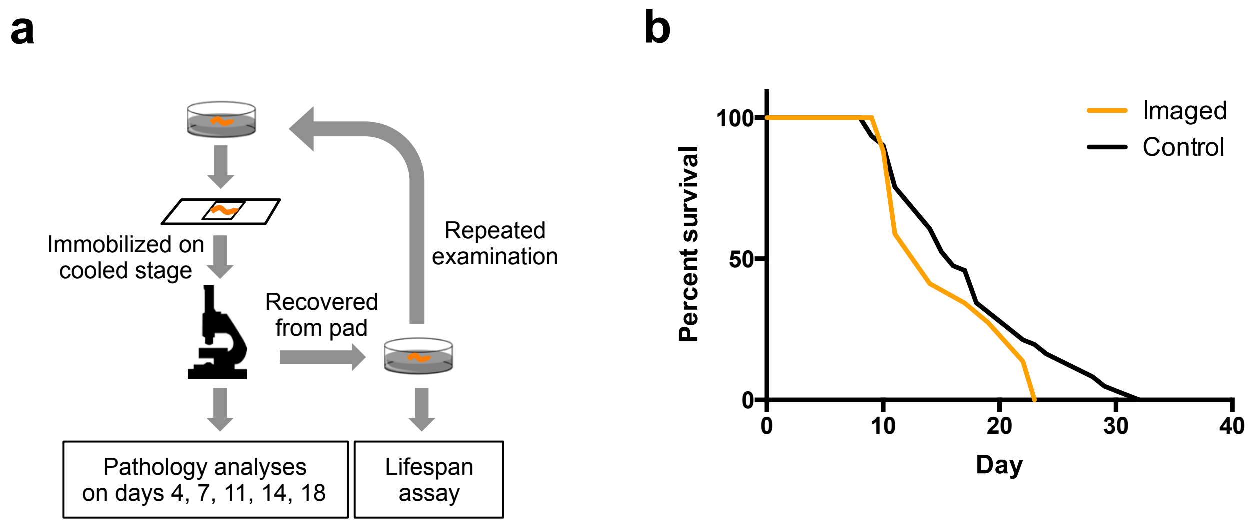

Supplementary Figure 1. Little effect on lifespan of repeated examination of worms using Nomarski microscopy.

(a) Longitudinal analysis of senescent pathology in individual worms, to test for correlations between severity of age-related pathologies and lifespan. **(b)** Imaged N2 hermaphrodites were examined on days 4, 7, 11, 14 and 18 of adulthood (Fig. 1a). Control worms were not removed from NGM plates. Median lifespan, imaged: 14 days (n=16); control: 16 days (n=61), p=0.16, log rank test. (Trials: 2)

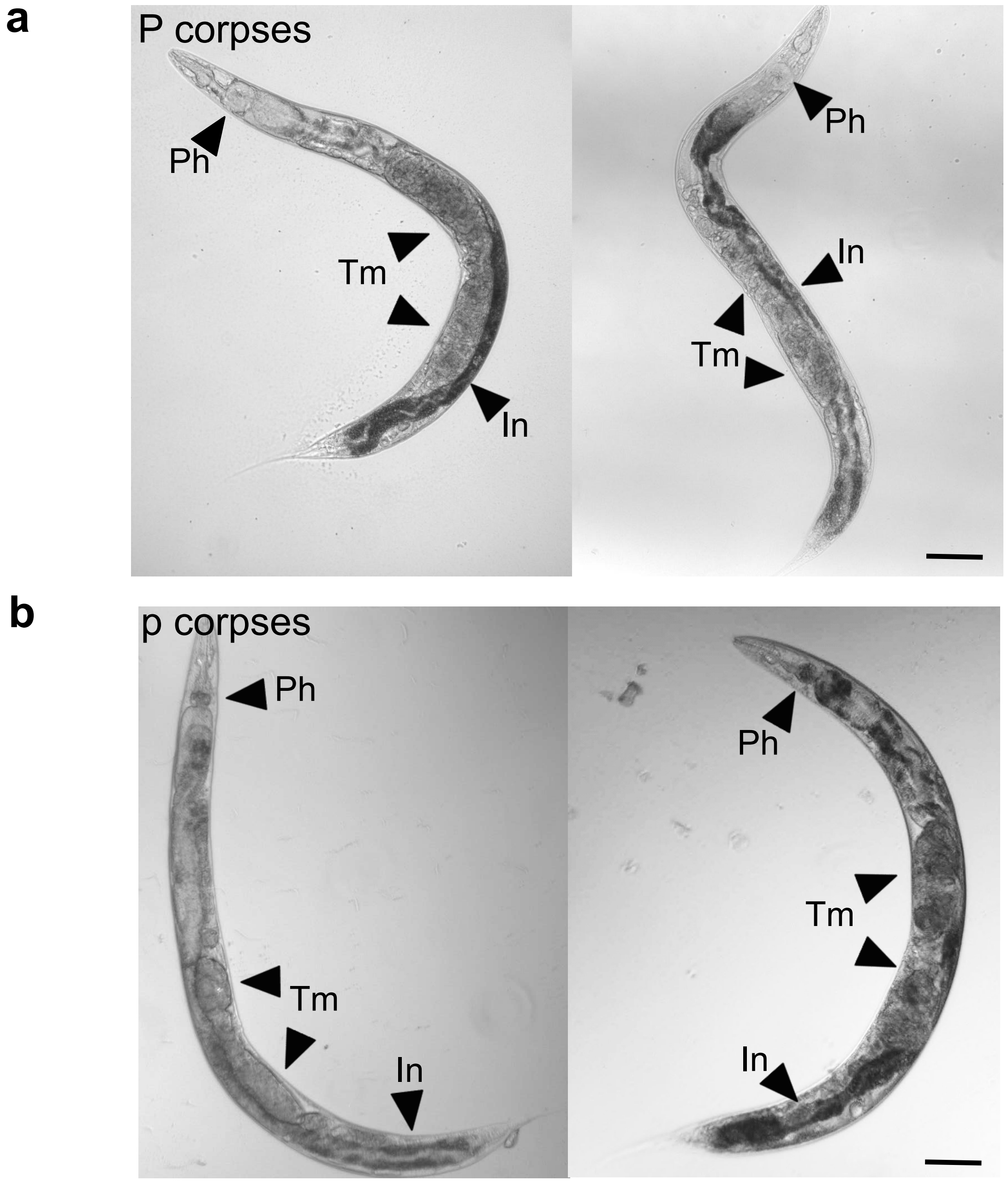

Supplementary Figure 2. Typical whole worm images of of P or p corpses.

(a) Nomarski images of worms that died on day 10 and 12 with P. **(b)** Nomarski images of worms that died on day 18 and 23 with p. Ph, pharynx; Tm, uterine tumours; In, intestine. Images captured at x100 magnification. Scale bars 80 µm.

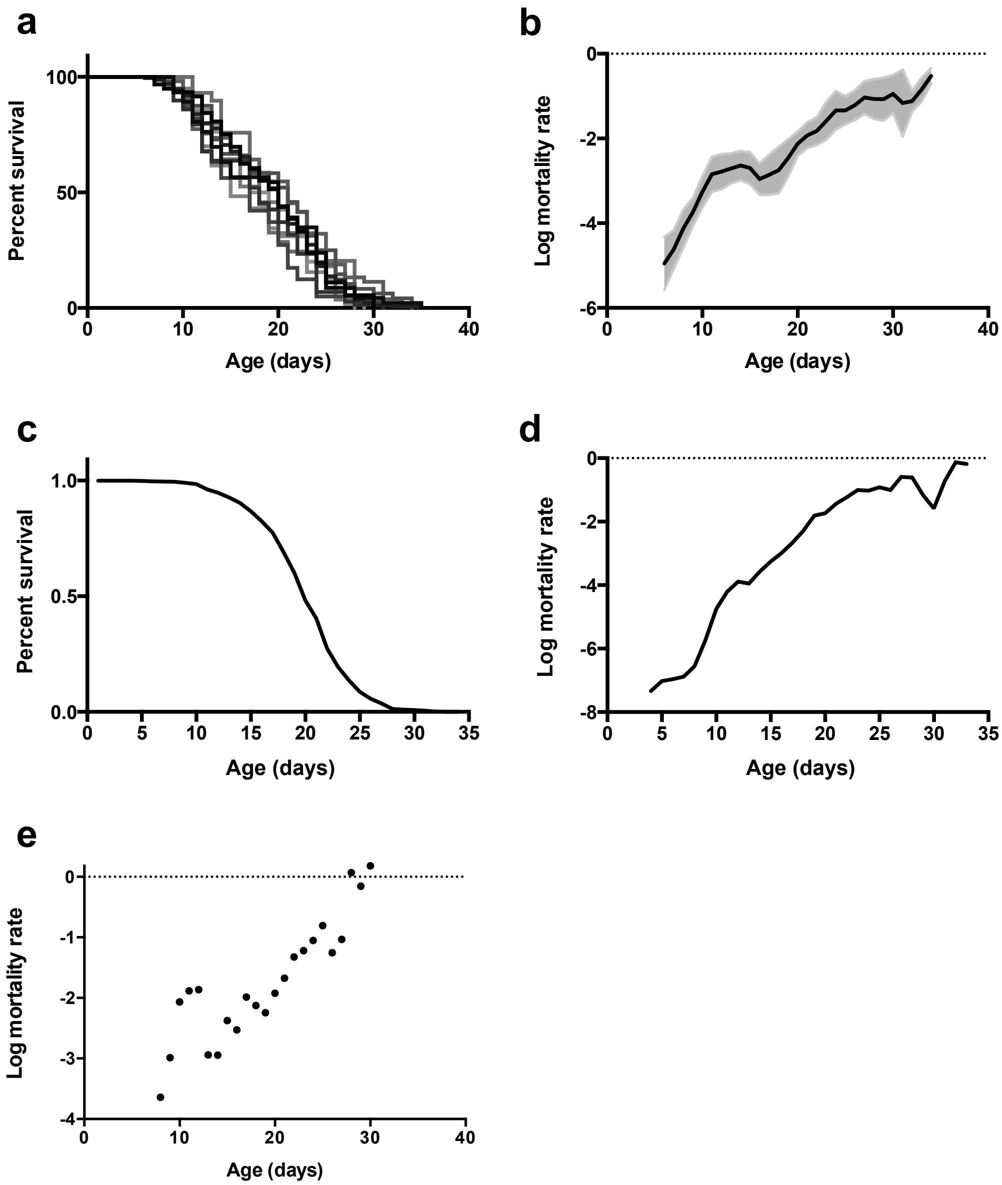

Supplementary Figure 3. Mortality analysis on N2 hermaphrodites.

(**a**) 11 independent trials of N2 hermaphrodite survival. (**b**) Log mortality analysis of survival data in (**a**). Solid line represents mean values, the shaded area represent 95% confidence interval. (**c**) Survival data combined from UMC and UCL. (**d**) Mortality data combined from both sites. Archive data from 9 (n=510) trials performed by D.G. at the University of Missouri-Columbia USA (UMC) (1994-6) and 10 (n=678) trials at University College London (UCL) (1998-2000)(20˚C). When adjusting for a 3-day difference in lifespan between UMC and UCL data and combining them, the change in the slope of log mortality against age is significantly different between the 4 observations points before day 11 and day 11, and day 11 and the 4 following observation time points (Welch t test p=0.00465, Bonferroni corrected for multiple testing). This is similar to mortality rate deceleration as previously described[1-5]. **(e)** Log mortality of wild-type survival data collected from one single, recent, large trial (n=585).

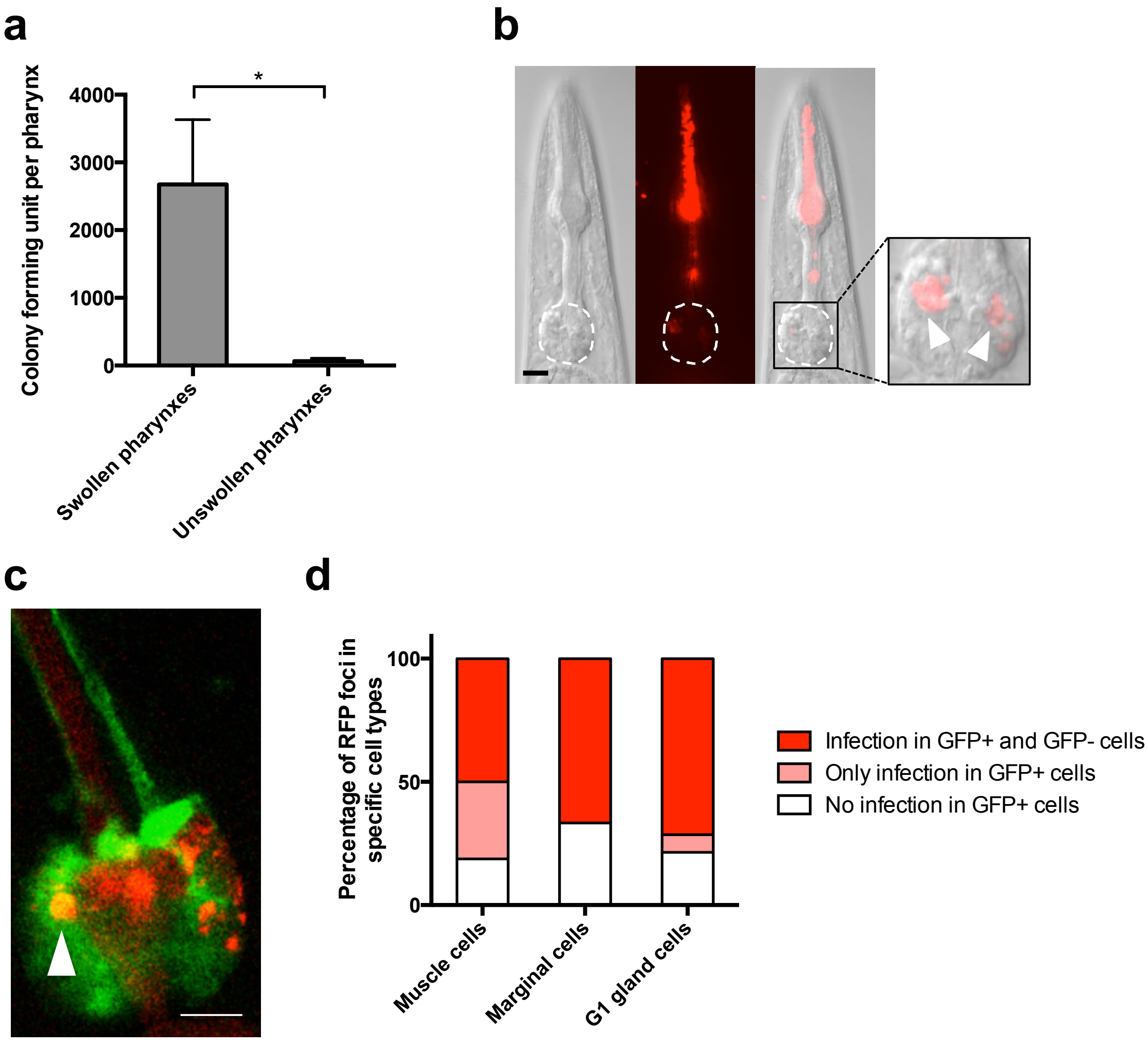

Supplementary Figure 4. Swollen pharynxes are caused by increased bacteria content.

(a) Colony forming units from excised, mascerated pharynxes from live day 10 or 11 adult worms with swollen or unswollen pharynxes. Swollen pharynxes, n=8; unswollen pharynxes, n=10, *p*=0.029 (*t*-test). N2 hermaphrodites, standard culture conditions. Data are mean±s.e.m. **(b)** p corpse from a worm fed *E. coli* expressing RFP which contains RFP inclusions in the pharynx (arrow heads). Scare bar 20 µm. **(c)** Confocal image of transgenic *C. elegans* expressing GFP in g1 gland cells, in early stage infection with RFP-tagged *E. coli*. Arrowhead, *E. coli* inclusion within gland cell. Scale bar 10 µm. **(d)** Frequency of overlap of GFP and RFP in each reporter strain. The greatest overlap is seen in muscle. Samples sizes for each reporter: pharyngeal muscle, 14; marginal cells, 12; gland cells 16.

a

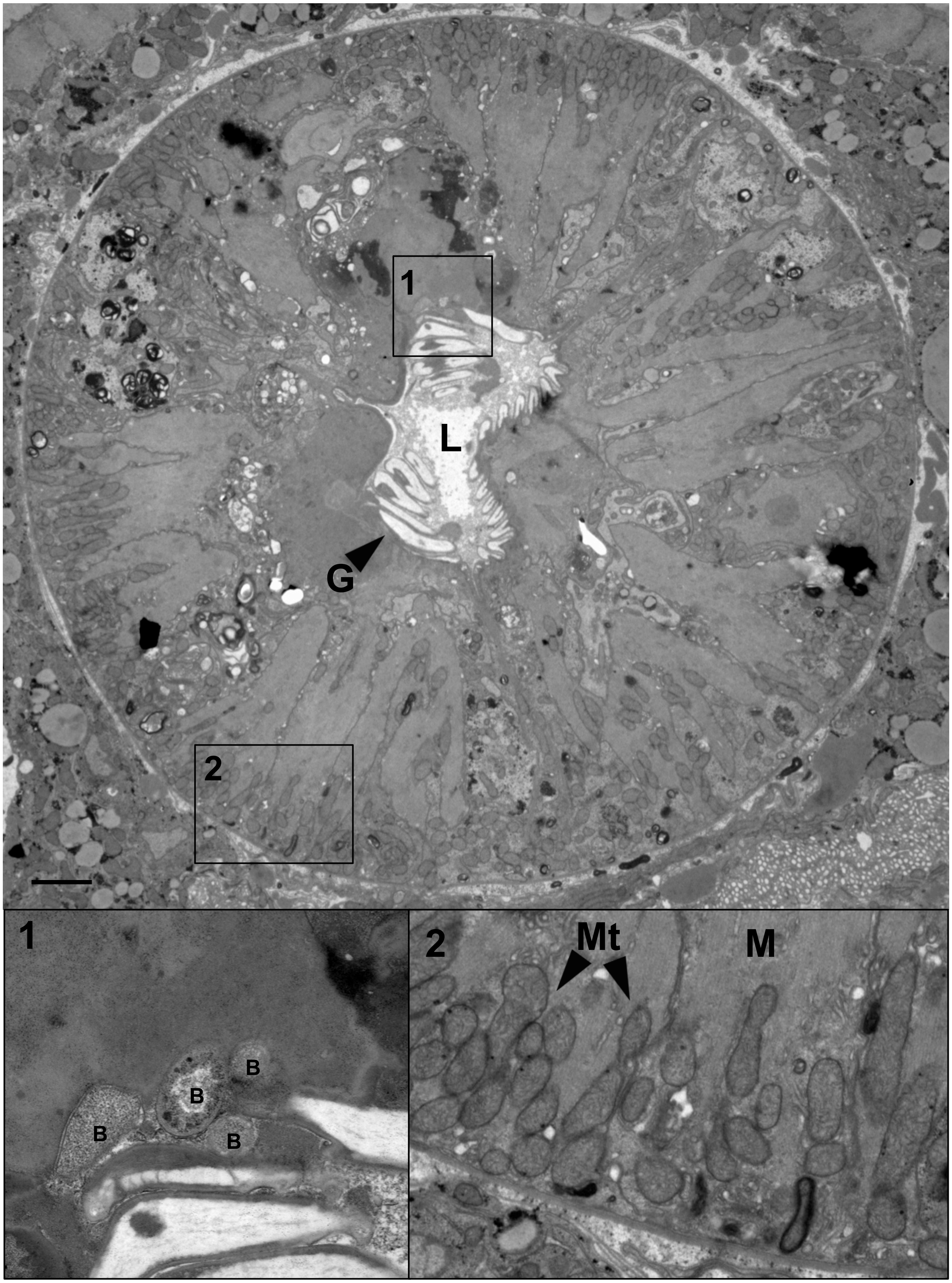

b

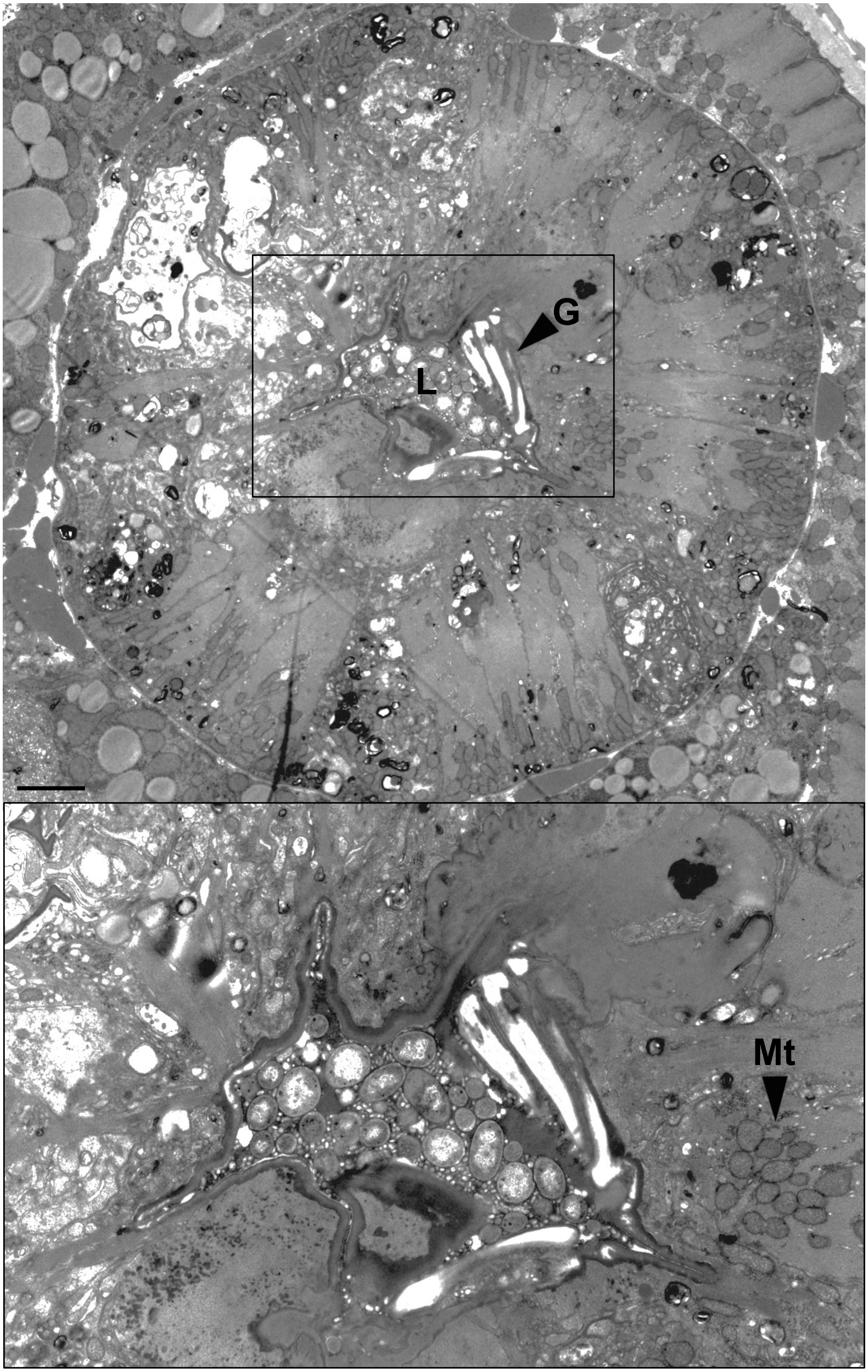

c

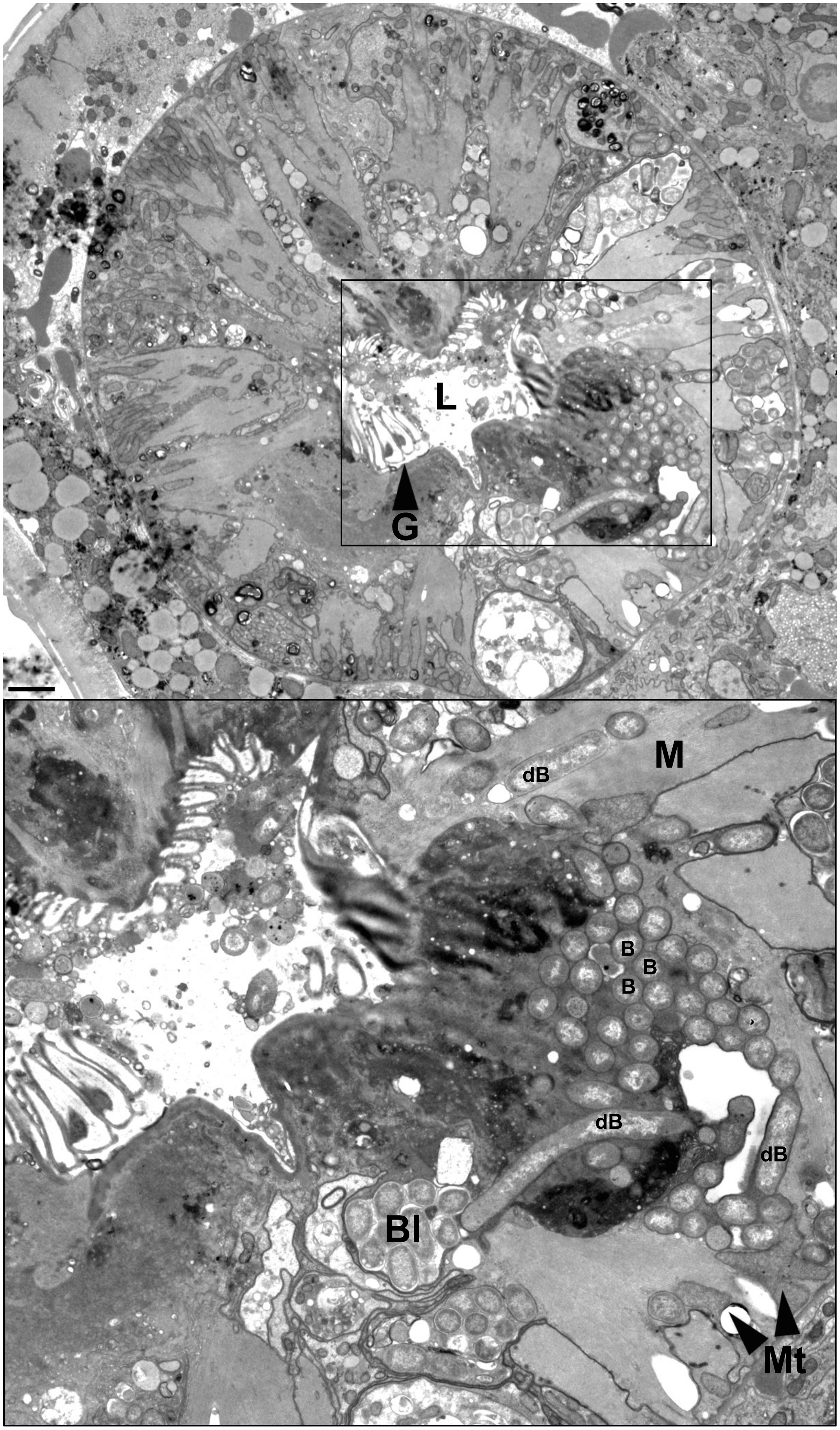

Supplementary Figure 5. Examination of pharyngeal pathology using electron microscopy.

N2 hermaphrodites on day 8 of adulthood, showing *E. coli* invasion of pharynx.
(a) Transmission electron micrograph showing pharynx with minor infection. Note small numbers of bacteria (B) (inset 1) apparently beyond the pharyngeal lumen (L), invading the pharyngeal cuticle at the base of the grinder (G). The bacteria are distinct in appearance from similarly sized mitochondria (Mt) in the muscle cells (M) (inset 2). Scale bar 2 µm. **(b)** A healthy pharynx with no apparent infection. Bacteria are contained within the lumen (L). Scale bar 2 µm. **(c)** A pharynx at an early stage of infection. Large numbers of bacteria (B) are seen within pharyngeal tissue near the grinder (G). The elongated appearance of some of the invading bacteria (dB) suggest that they are dividing within pharyngeal tissue. An inclusion is present where bacteria are contained within a double membrane structure (BI). Scale bar 2 µm.
A total of 3 swollen and 2 unswollen pharynxes were examined.

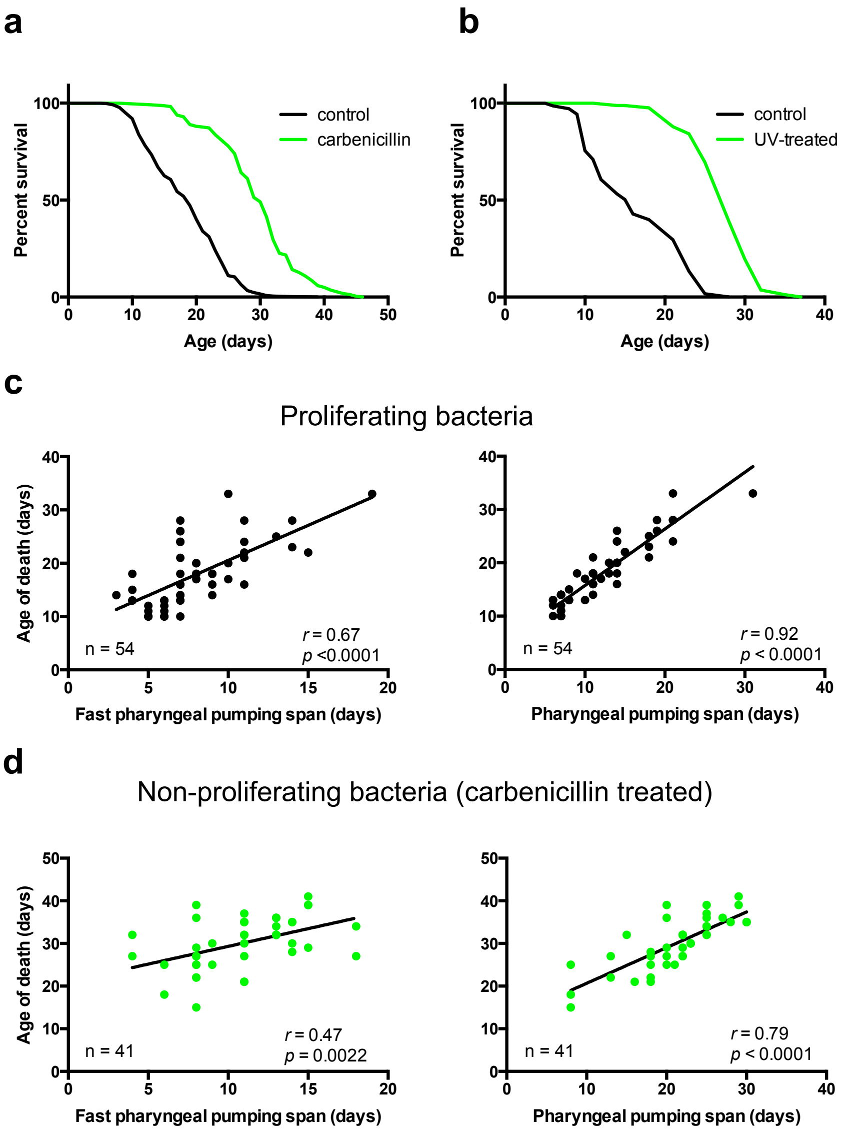

Supplementary Figure 6. Effect of inhibition of *E. coli* proliferation on lifespan and pumping span.

(a) Blocking *E. coli* proliferation using the antibiotic carbenicillin extends worm lifespan by 60% (p<0.0001, log rank test, trials: 4). **(b)** Blocking *E. coli* proliferation using UV-irradiation extends lifespan by 67% (p<0.0001 log rank test, trials: 2), consistent with previous observations[6,7] (Supplementary Table 3; Supplementary dataset 1). **(c)** Correlation between pumping span and age of death in worms aged on proliferating *E. coli*. (n=54) **(d)** Worms aged on non-proliferating *E. coli* (treated with carbenicillin) (n=41). Statistical comparison of the relationship between pharyngeal pumping span or fast pharyngeal pumping span and age of death using a linear model revealed the relationship between the two (pumping and age at death) showed a near significant alteration in the presence of carbenicillin (pharyngeal pumping span, control vs. carbenicillin p=0.056; fast pharyngeal pumping span control vs. carbenicillin p=0.13). This data suggests that the occurrence of P death partially explains the correlation between pharyngeal pumping rate and lifespan. (Trials: 2).

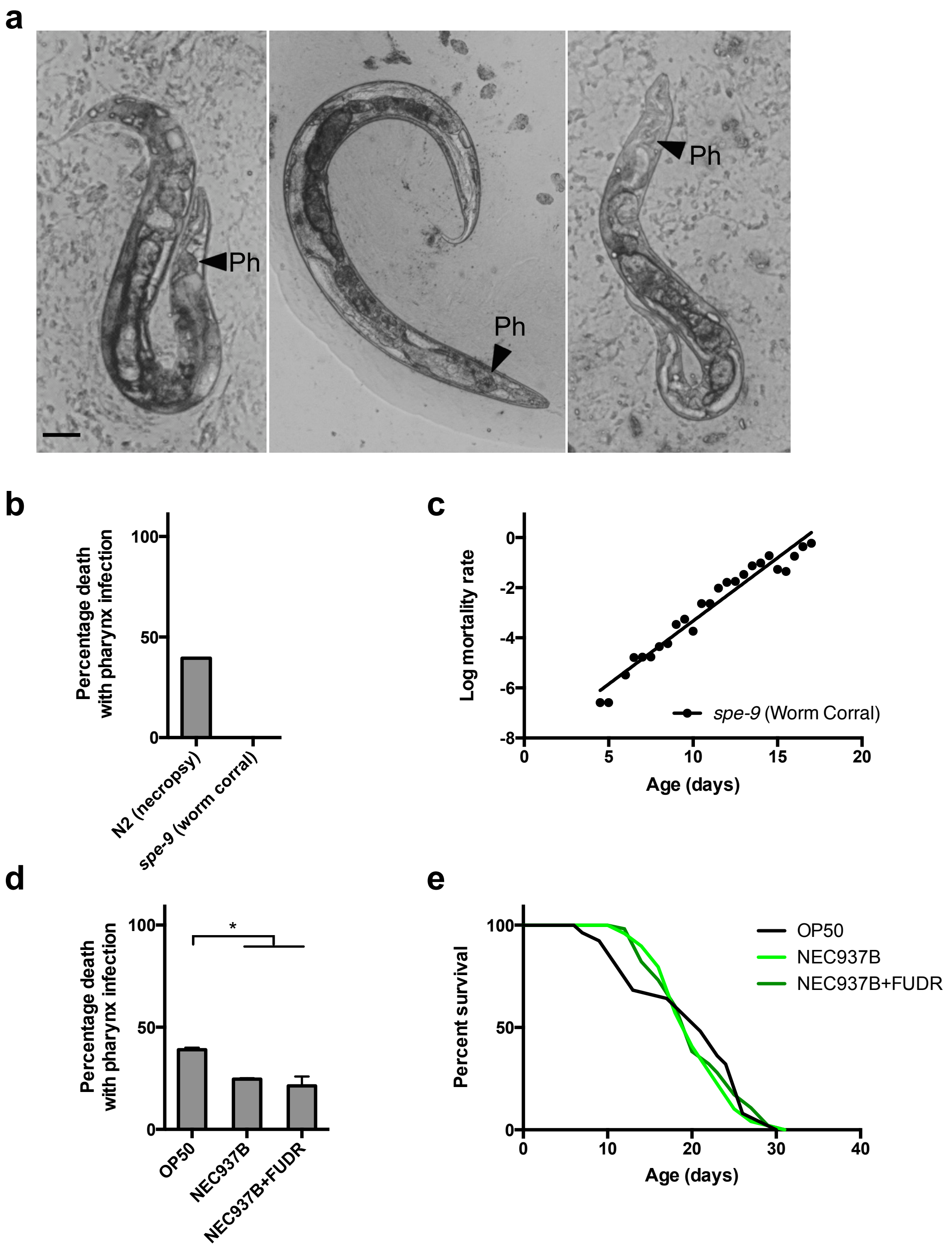

Supplementary Figure 7. Automated lifespan conditions reduce or eliminate P death.

(**a**) Images of worm corpses from worm corral trials, representative of 725 worms examined. Pharynxes (Ph) do not show any evidence of swelling; instead all worms appeared to undergo p death. Scale bar 80 µm. (**b**) Worm Corral culture condition eliminates P death. N2 data are from Fig. 1d. (**c**) Log mortality of *spe-9* survival data collected in worm corral. (**d**) Reduced frequency of P when worms were cultured on *E. coli* strain NEC937 B. (Trial: 1). **(e)** Survival curve of wild-type worms on OP50 and NEC937 B bacteria (Trial: 1).

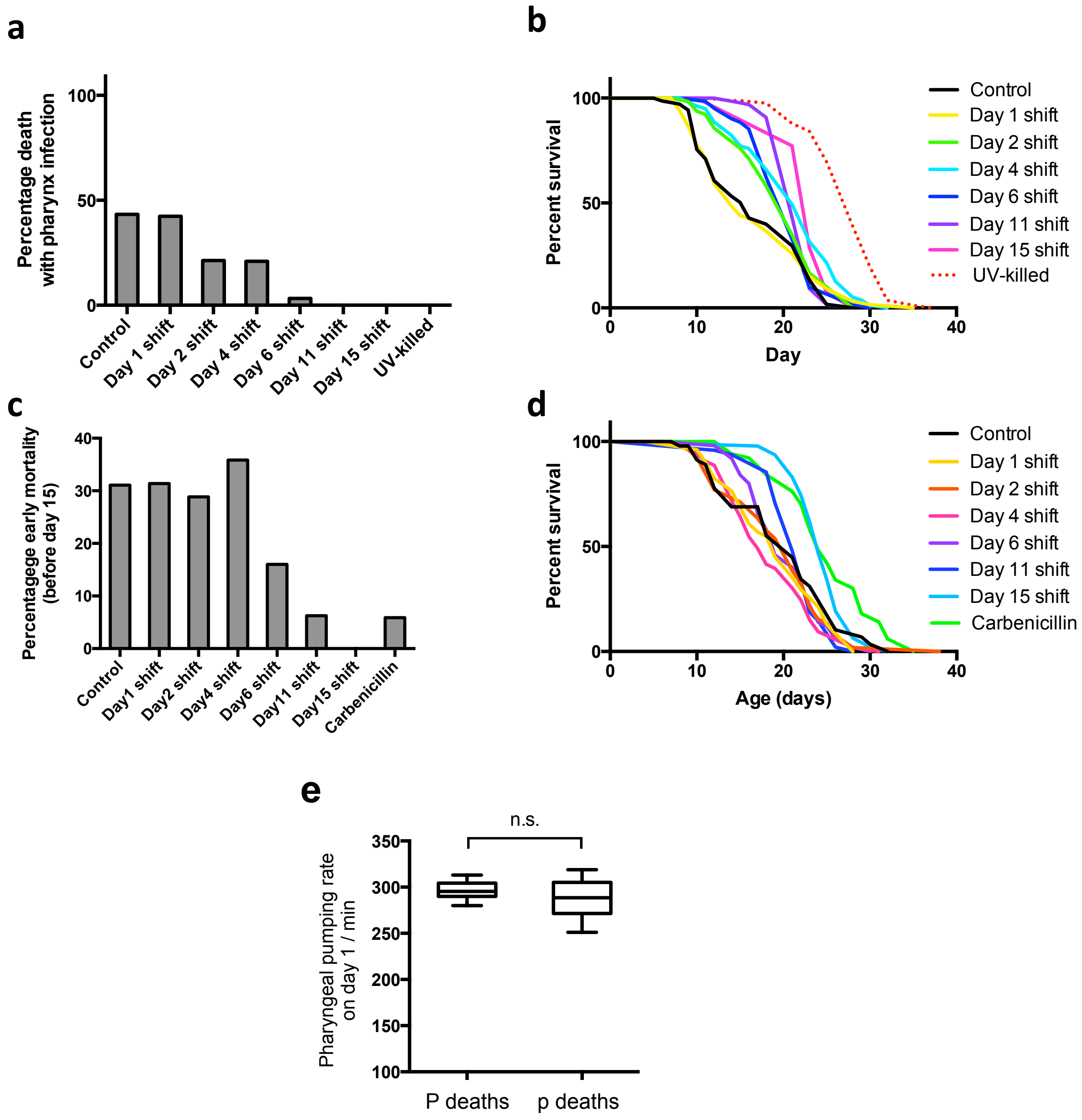

Supplementary Figure 8. Effects of non-proliferating *E. coli* and pharyngeal pumping rate in early life.

(a) Effect on frequency of P deaths from shifts off UV-irradiated *E. coli*. (Trial 1). **(b)** Effect on survival. Control, maintained on live *E. coli* throughout life. UV, maintained on irradiated *E. coli* throughout life. These results show that *E. coli* exposure in early life is critical for development of pathology leading to P death. (Trial: 1). **(c)** Effect on early mortality (defined as pre-day 15) from shifts off carbenicillin treated bacteria. (Trials: 2). **(d)** Effect on survival from shifts off carbenicillin treated bacteria. Graph is the result of one representative trial. (Trials: 2). (**e**) Pharyngeal pumping rate on day 1 of adulthood of worms that undergo either P or p death (P death n=14, p death n=10) (p=0.25, t test). Data are mean±s.e.m. (Trials: 2).

a

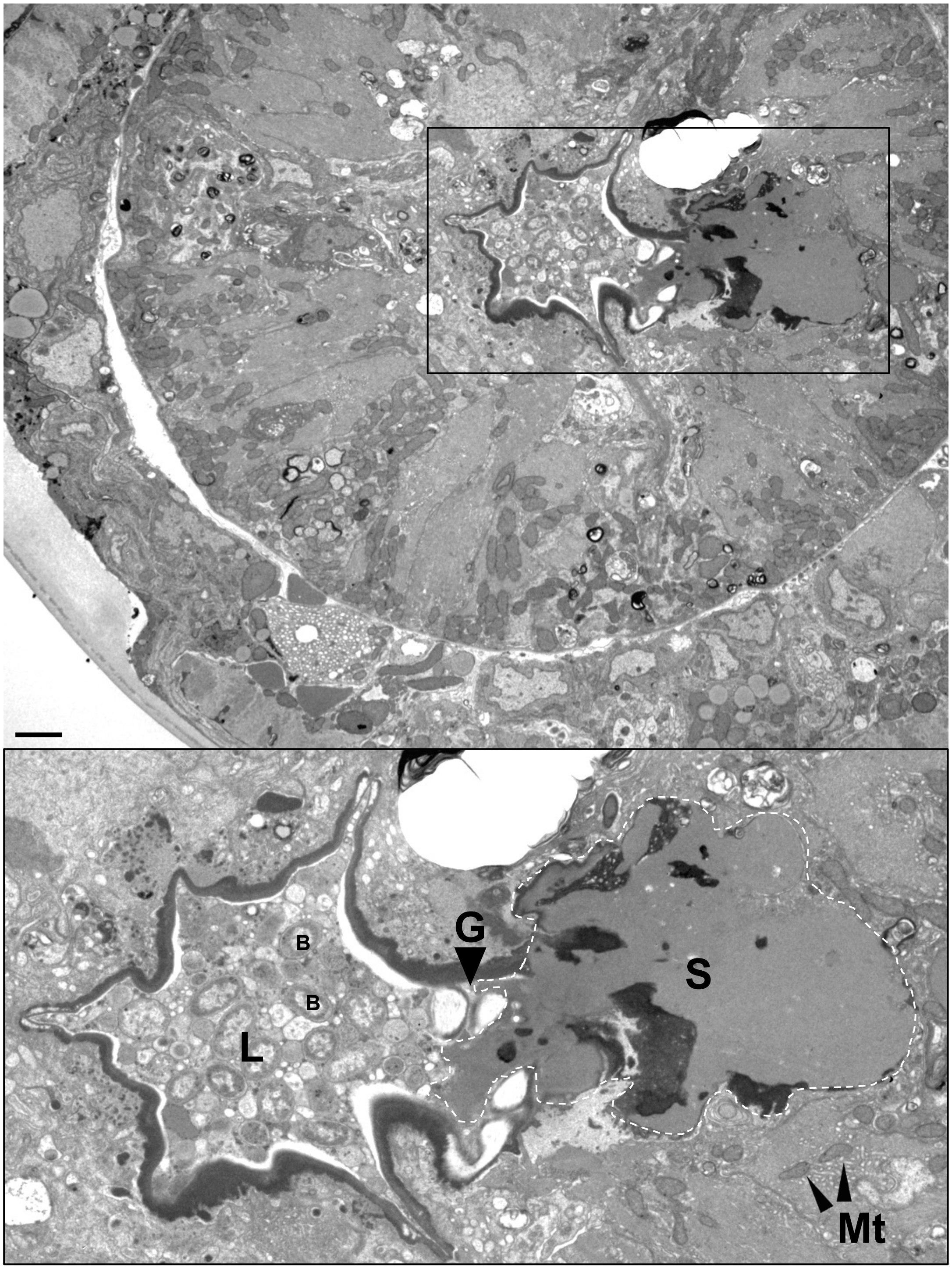

b

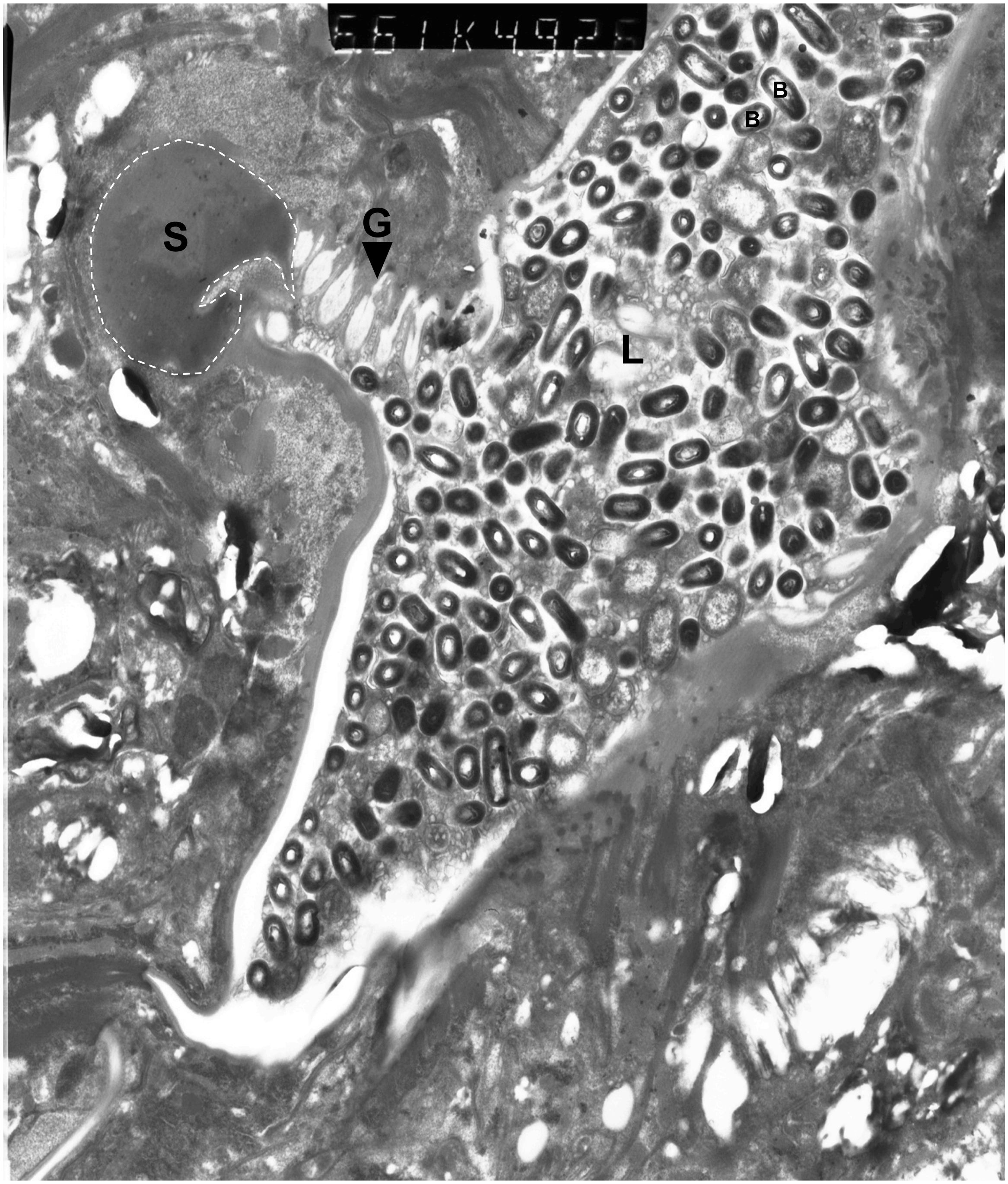

Supplementary Figure 9. Evidence of electron-dense scarring near the grinder of the pharynx in aged worms.

(a) Cross section of the pharyngeal grinder of a day 8 adult. Scale bar 2μm. **(b)** Image from WormImage (Hall Lab, N824_4926) of a day 7 adult. G grinder, L lumen, B bacteria, Mt mitochondria, S scar (outlined in white).

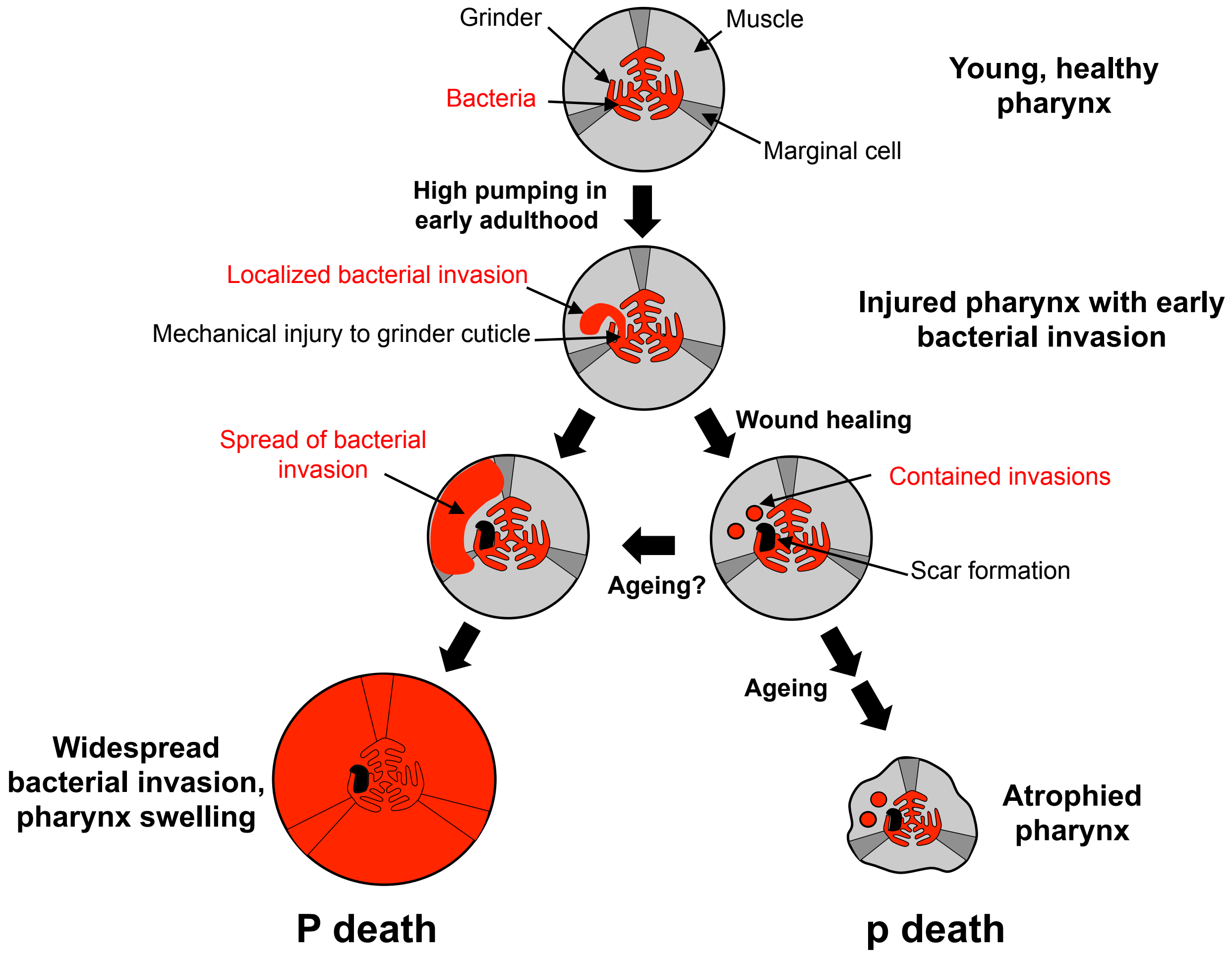

Supplementary Figure 10. Model for mechanism determining P death.
According to this model, high rate of pharyngeal pumping in early adulthood leads to mechanical damage (mechanical senescence) to the pharyngeal cuticle. This perforates the cuticle, allowing minor bacterial invasion of pharyngeal tissue. The invasion could spread further leading to widespread infection and pharyngeal swelling (P death). Alternatively initial invasion could be contained and wound healing leads to closure of cuticular perforation. *E. coli* inclusions in the pharynx may lead to major infection later in life in some worms, due to the effects of ageing. Why some worms succumb to such infection and others not remains to be determined.

Several observations support this model. (a) Blocking *E. coli* proliferation prevents P death. (b) Reducing pharyngeal pumping rate prevents P death. (c) Carbenicillin and UV shift experiments show that the critical period for exposure to *E. coli* to lead to P death is early adulthood, when pharyngeal pumping rate is high. (d) Confocal microscopy of worms fed with RFP-labelled *E coli*, and TEM analysis shows initial invasion of pharyngeal tissue near to the pharyngeal lumen. (e) Electron microscopy shows major scarring of the pharyngeal cuticle in the grinder region. (f) Epifluorescence microscopy of worms fed with RFP-labelled *E. coli* shows RFP-positive inclusions within the pharynx. The mechanisms leading to pharyngeal atrophy in p death remain unexplored.

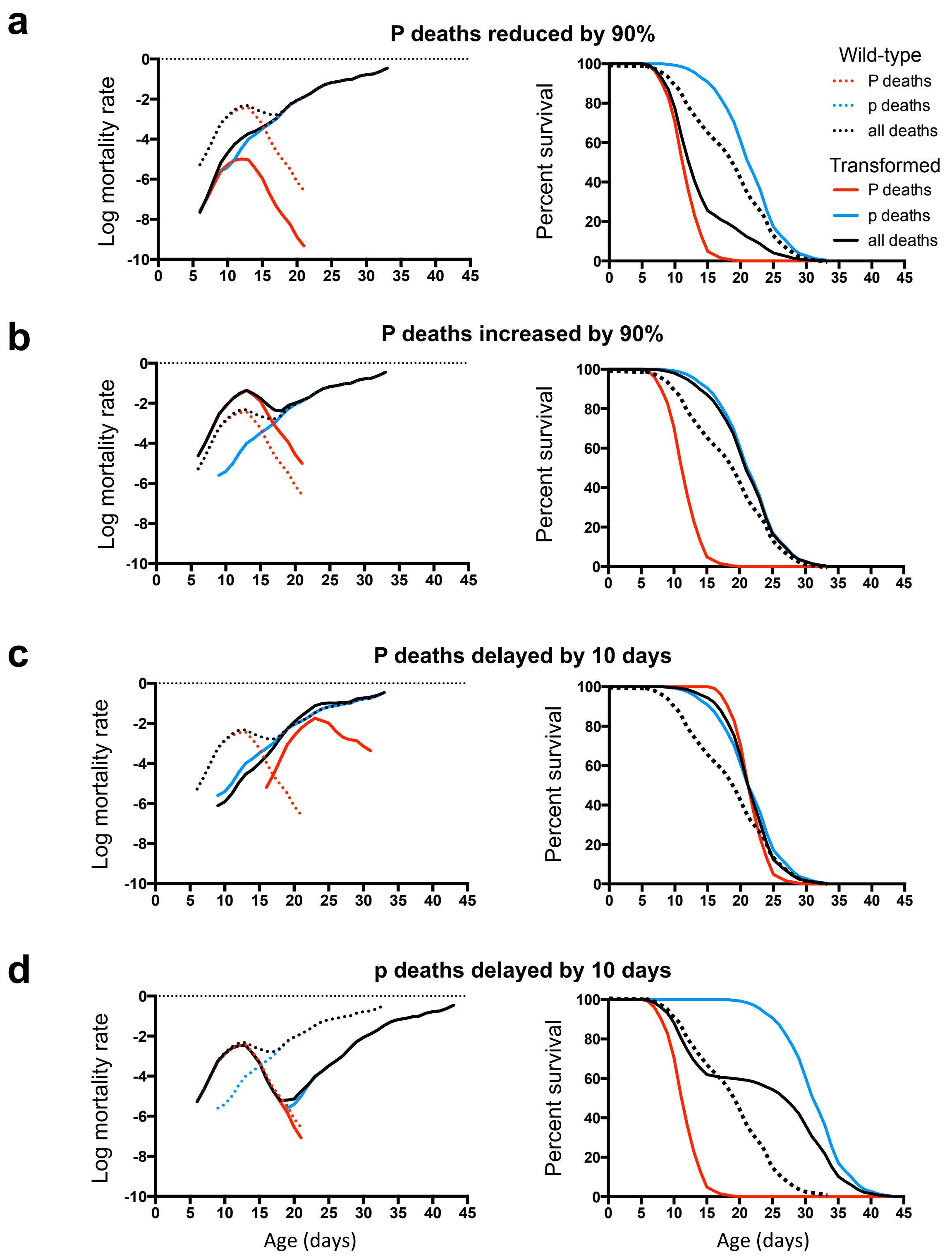

Supplementary Figure 11. Mortality and survival plots of theoretical changes to frequency or timing of P or p deaths.
(a) P deaths decreased by 90%. **(b)** P deaths increased by 90%. **(c)** P deaths delayed by 10 days. **(d)** p deaths delayed by 10 days. Data includes whole population (black) and P (red) or p (blue) deaths as in Fig. 4. Dotted lines, wild-type data; solid lines, data after specified transformation.

	Day 4		Day 7		Day 11		Day 14	
	R square	*p* value	R square	*p* value	R square	*p* value	R square	*p* value
**Pharyngeal deterioration**	0.0019	0.8723	0.3963	0.0090	0.7826	0.0035	0.2667	0.2943
**Gonadal atrophy**	0.0108	0.7130	0.4122	0.0099	0.3706	0.1468	0.1938	0.5598
**Intestinal atrophy**	0.4443	0.0067	0.0013	0.9003	0.5584	0.0331	0.4396	0.2226
**Yolk pool accumulation**	0.1781	0.1171	0.2411	0.0631	0.3031	0.2003	0.1003	0.6035
**Uterine tumor size**	0.1837	0.0977	0.5185	0.0017	0.0014	0.9298	0.0741	0.6578

Supplementary Table 1. Summary of the correlation between age at death and severity of various age-related pathologies.

Individual worms were imaged at certain time points and then recovered for a survival assay. Data was analyzed using linear regression. Correlations that were significant have been highlighted in green.

	all deaths	P deaths	p deaths
**Number of values**	622	227	395
**Mean**	18.50	12.36	22.04
**Standard deviation**	6.184	3.102	4.527
**Coefficient of variation**	33%	25%	21%
**Sum of squares**	23786	2184	8095
**Explained variance**		9%	34%

Supplementary Table 2. Summary of N2 all deaths, P deaths and p deaths subpopulations.

Variances within P and p subpopulations account for 9% and 34% of the total variance in lifespan, respectively. The difference between the two subpopulations explains 57% of the total variance in lifespan.

**Strain/condition**	**Number of deaths/censored**	**Mean [median] life span (days)**	**% change vs. control**	***p* vs. control (log rank)**
N2 (control)	**[C] 157/42**<br>[1] 67/18<br>[2] 90/24	**17.6 [18]**<br>16.6 [16]<br>18.3 [19]		
N2 (UV-killed)	**[C] 157/44**<br>[1] 83/18<br>[2] 74/26	**26.6 [28]**<br>27.7 [28]<br>25.5 [25]	**+51.1 [+56]**<br>+66.9 [+75]<br>+39.3 [+32]	**<0.0001**<br><0.0001<br><0.0001
N2 (control)	**[C] 419/75**<br>[1] 42/8<br>[2] 277/51<br>[3] 59/6<br>[4] 41/10	**17.7 [17]**<br>17.9 [17]<br>18.8 [20]<br>17.2 [15]<br>18.7 [18]		
N2 (carbenicillin)	**[C] 225/13**<br>[1] 50/0<br>[2] 68/1<br>[3] 67/9<br>[4] 40/3	**29.4 [30]**<br>30.4 [32]<br>30.5 [30]<br>29.7 [29]<br>27.1 [27]	**+66.1 [+76]**<br>+69.8 [+88]<br>+62.0 [+50]<br>+73.0 [+93]<br>+45.2 [+50]	**<0.0001**<br><0.0001<br><0.0001<br><0.0001<br><0.0001

Supplementary Table 3. Effect of non-proliferative bacteria on lifespan.

Trials were performed at 20˚C. [C] is combined data from all trials, [n] is trial number.

**Strain/condition**	**Number of deaths/censored**	**Mean [median] life span (days)**	**% change vs. control**	***p* vs. control (log rank)**
N2 (control)	**[C] 95/33**<br>[1] 37/19<br>[2] 58/14	**19.1 [18]**<br>19.5 [21]<br>18.8 [18]		
N2 (day 1 shift off carbenicillin)	**[C] 110/15**<br>[1] 50/7<br>[2] 60/8	**19.0 [19]**<br>19.0 [19]<br>19.1 [18]	**-0.5 [+5.6]**<br>-2.6 [-9.5]<br>+1.6 [0.0]	**0.9469**<br>0.4215<br>0.4972
N2 (day 2 shift off carbenicillin)	**[C] 111/14**<br>[1] 52/5<br>[2] 59/9	**18.9 [21]**<br>19.2 [21]<br>18.6 [21]	**-1.0 [+16.7]**<br>-1.5 [0.0]<br>-1.1 [+16.7]	**0.8428**<br>0.6741<br>0.6125
N2 (day 4 shift off carbenicillin)	**[C] 109/9**<br>[1] 54/1<br>[2] 55/8	**19.1 [18]**<br>18.1 [17]<br>20.0 [21]	**0.0 [0.0]**<br>-7.2 [-19.0]<br>+6.4 [+16.7]	**0.9957**<br>0.1169<br>0.1600
N2 (day 6 shift off carbenicillin)	**[C] 107/8**<br>[1] 50/4<br>[2] 57/4	**20.7 [21]**<br>20.0 [19]<br>21.2 [21]	**+8.4 [+16.7]**<br>+2.6 [-9.5]<br>+12.8 [+16.7]	**0.1291**<br>0.7477<br>0.0115
N2 (day 11 shift off carbenicillin)	**[C] 107/1**<br>[1] 48/0<br>[2] 59/1	**21.3 [21]**<br>21.1 [21]<br>21.5 [21]	**+11.5 [+16.7]**<br>+8.2 [0.0]<br>+14.4 [+16.7]	**0.0598**<br>0.9389<br>0.0092
N2 (day 15 shift off carbenicillin)	**[C] 101/1**<br>[1] 47/0<br>[2] 54/1	**23.5 [24]**<br>24.3 [24]<br>22.8 [23]	**+23.0 [+33.3]**<br>+24.6 [+14.3]<br>+21.3 [+27.8]	**<0.0001**<br>0.0037<br><0.0001
N2 (carbenicillin)	**[C] 116/13**<br>[1] 50/8<br>[2] 66/5	**27.2 [28]**<br>24.8 [24]<br>29.0 [30]	**+42.4 [+55.6]**<br>+27.2 [+14.3]<br>+54.3 [+66.7]	**<0.0001**<br>0.0002<br><0.0001
N2 (control)	[1] 67/18	16.6 [16]		
N2 (day 1 shift off UV-killed)	[1] 66/9	16.6 [14]	0.0 [-12.5]	0.7922
N2 (day 2 shift off UV-killed)	[1] 61/14	19.6 [21]	+18.1 [+31.25]	0.0233
N2 (day 4 shift off UV-killed)	[1] 62/13	19.2 [21]	+15.7 [+31.25]	0.0979
N2 (day 6 shift off UV-killed)	[1] 61/14	20.3 [21]	+22.3 [+31.25]	0.0201
N2 (day 11 shift off UV-killed)	[1] 65/10	21.7 [21]	+30.7 [+31.25]	0.0010
N2 (day 15 shift off UV-killed)	[1] 64/11	22.6 [23]	+36.1 [+43.8]	<0.0001
N2 (UV-killed)	[1] 83/18	27.7 [28]	+66.9 [+75.0]	<0.0001

Supplementary Table 4. Effect of shifting worms off non-proliferative bacteria on lifespan. Trials were performed at 20˚C. [C] is combined data from all trials, [n] is trial number.

**Strain/ condition**	**Number of deaths/ censored**	**Mean [median] lifespan (days)**	**% change vs. N2 control**	***p* vs. control (log rank)**
N2 (control)	**[C] 109/31**<br>[1] 46/24<br>[2] 63/7	**18.6 [19]**<br>18.8 [18]<br>18.4 [19]		
eat-2(ad1116)	**[C] 109/23**<br>[1] 55/15<br>[2] 54/8	**21.4 [21]**<br>21.2 [21]<br>21.6 [23]	**+15.1 [+10.5]**<br>+12.8 [+16.7]<br>+17.4 [+21.1]	**0.0009**<br>0.0440<br>0.0039

Supplementary Table 5. Effect of *eat-2* on lifespan.

Trials were performed at 20˚C. [C] is combined data from all trials, [n] is trial number.

Strain/condition	Number of deaths/censored	Mean [median] life span (days)	% change vs. control	*p* vs. control (log rank)
glp-1(e2141)<br>L4 shift to 25ºC	**[C] 125/1**<br>[1] 59/0<br>[2] 66/1	**11.5 [9]**<br>11.7 [10]<br>11.3[9]		
glp-1(e2141)<br>egg shift to 25ºC	**[C] 176/9**<br>[1] 78/7<br>[2] 98/2	**15.8 [10]**<br>16.1 [10]<br>15.5[10]	**+37.4 [11]**<br>+37.8 [0]<br>+37.2 [11]	**<0.0001**<br><0.0001<br><0.0001

Supplementary Table 6. Effect of shifting *glp-1* mutants to non-permissive temperature at either egg or L4 stage on lifespan.

Worms were raised at 15˚C until shifted. Lifespans were performed at 25˚C. [C] is combined data from all trials, [n] is trial number.

Strain/condition	Number of deaths/censored	Mean [median] life span (days)	% change vs. control	*p* vs. control (log rank)
N2 (control)	**[C] 99/8**<br>[1] 48/3<br>[2] 51/5	**19.76 [21]**<br>19.85 [21]<br>19.67 [21]		
ced-1(e1735)	**[C] 119/8**<br>[1] 67/3<br>[2] 52/5	**15.63 [14]**<br>15.82 [14]<br>15.38 [15]	**-20.9 [-33]**<br>-20.3 [-33]<br>-21.8 [-29]	**<0.0001**<br><0.0001<br><0.0001

Supplementary Table 7. Effect of *ced-1* mutants on lifespan.

Lifespans were performed at 20˚C. [C] is combined data from all trials, [n] is trial number.

Supplementary References:

1 Wu, D., Rea, S. L., Yashin, A. I. & Johnson, T. E. Visualizing hidden heterogeneity in isogenic populations of *C. elegans*. *Exp. Gerontol.* **41**, 261-270 (2006).

2 Vaupel, J. W., Johnson, T. E. & Lithgow, G. J. Rates of mortality in populations of *Caenorhabditis elegans*. *Science* **266**, 826 (1994).

3 Johnson, T. E., Wu, D., Tedesco, P., Dames, S. & Vaupel, J. W. Age-specific demographic profiles of longevity mutants in *Caenorhabditis elegans* show segmental effects. *J. Gerontol.* **56**, B331-339 (2001).

4 Brooks, A., Lithgow, G. J. & Johnson, T. E. Mortality rates in a genetically heterogeneous population of *Caenorhabditis elegans. Science* **263**, 668-671 (1994).

5 Vanfleteren, J. R., De Vreese, A. & Braeckman, B. P. Two-parameter logistic and Weibull equations provide better fits to survival data from isogenic populations of *Caenorhabditis elegans* in axenic culture than does the Gompertz model. *J. Gerontol.* **53**, B393-403 (1998).

6 Garigan, D. *et al.* Genetic analysis of tissue aging in *Caenorhabditis elegans*: a role for heat-shock factor and bacterial proliferation. *Genetics* **161**, 1101-1112 (2002).

7 Gems, D. & Riddle, D. L. Genetic, behavioral and environmental determinants of male longevity in *Caenorhabditis elegans*. *Genetics* **154**, 1597-1610 (2000).
